# Loss of *SPATULA* Function Partially Suppresses Root Growth and Meristem Organization Defects in *GCN5* Mutants in *Arabidopsis thaliana*

**DOI:** 10.3390/plants15203181

**Published:** 2026-10-16

**Authors:** Stylianos Poulios, Christos Tersenidis, Sofia Sidiropoulou, Christina Maria Staikoglou, Maria Aikaterini Kaftantzi, Konstantinos E. Vlachonasios

**Affiliations:** 1 Department of Botany, School of Biology, Aristotle University of Thessaloniki, GR-54124 Thessaloniki, Greece; spoulios@bio.auth.gr (S.P.); ctersen@bio.auth.gr (C.T.); sofisidi@bio.auth.gr (S.S.); staikogl@bio.auth.gr (C.M.S.); mkaftant@bio.auth.gr (M.A.K.); 2 Natural Products Research Centre of Excellence (NatPro-AUTh), Center of Interdisciplinary Research and Innovation of Aristotle University of Thessaloniki (CIRIAUTh), GR-57001 Thessaloniki, Greece

**Keywords:** quiescent center, root meristem, root growth, GCN5, SPT

## Abstract

GENERAL CONTROL NON-DEREPRESSIBLE 5 (GCN5) is a histone acetyltransferase that plays a central role in regulating plant development and responses to environmental stimuli. In root development, GCN5 promotes the maintenance of the stem cell niche and proliferation of transit-amplifying cells. SPATULA (SPT) is a basic helix-loop-helix (bHLH) transcription factor with established roles in gynoecium and carpel development, as well as broader functions in plant growth. In roots, SPT negatively regulates quiescent center size, root meristem size, and overall root growth, without substantially affecting root patterning or cell-type differentiation. To investigate a potential genetic interaction between *SPT* and *GCN5* in root meristem development, we generated and characterized *spt*;*gcn5* double mutants. Notably, loss of *SPT* partially suppressed some of the developmental defects associated with *gcn5*, including abnormalities in root meristem size, meristem organization, and root growth. These data suggest that a complicated genetic interaction between SPT and GCN5 regulates important aspects of root meristem growth and organization.

## 1. Introduction

The root is the plant organ responsible for the vital functions of nutrient and water uptake, as well as plant mechanical support and anchoring. Other important root functions include the storage of nutrients and interaction with the soil microbiome [1]. The root apex of *Arabidopsis thaliana* consists of four distinct zones of growth activities: the meristematic, the transition, the elongation and the differentiation zone. The meristematic zone (Root Apical Meristem, RAM) comprises all mitotically active cells. Followed by the transition zone, where cells prepare for fast elongation through physiological changes, such as the development of a central vacuole, polarization of the cytoskeleton and cell wall remodeling. In the elongation zone, the cells undergo fast anisotropic cell growth before entering the differentiation zone, where they follow their terminal differentiation pathway [2]. RAM organization in *Arabidopsis thaliana* is established during embryogenesis, and the pattern of cell divisions in the embryo gives rise to the various initials within the root pro-meristem [3]. The hypophysis-derived quiescent center (QC) cells are surrounded by the root initial cells, and together they constitute the stem cell niche (SCN). These stem cells give rise to distinct root cell lineages, including the stele, cortex and endodermis, epidermis and lateral root cap, and columella [4,5]. SCN maintenance in the *Arabidopsis thaliana* root is essential for the coordinated proliferation, differentiation, and development of root cell types. Several key transcription factors and their related pathways modulate SCN specification and maintenance, such as WUSCHEL-RELATED HOMEOBOX 5 (WOX5), PLETHORAs (PLTs) [6,7], SHORT-ROOT (SHR) and SCARECROW (SCR) [8,9,10,11].

In addition to transcription factors, the regulation of root growth and development involves the action of plant hormones and chromatin remodeling [12,13]. GENERAL CONTROL NON-DEREPRESSIBLE 5 (GCN5) is a histone acetyltransferase that regulates a multitude of developmental processes and responses to abiotic and biotic factors in plants [14,15,16,17]. In root development, *GCN5* promotes SCN maintenance and transit-amplifying cell proliferation via the PLETHORA (PLT) pathway [15]. GCN5 seems to boost rather than establish *PLT1* and *PLT2* expression, sustaining the higher levels required to maintain the stem cell profile. Additionally, GCN5 mediates proliferation of the transit-amplifying cells, possibly in part by regulating *CYCLINB1;1* (*CYCB1;1*) and *CYCLINB1;3* (*CYCB1;3*) expression [13,15]. GCN5 also works in the root as a positive regulator of auxin biosynthesis and transport [18] and cytokinin homeostasis [19]. SPATULA (SPT) is a basic helix-loop-helix (bHLH) transcription factor involved in gynoecium and carpel development as well as many other aspects of plant growth [20,21]. SPT in root development suppresses QC size, root meristem size, and root length in general [22]. *SPT* affects meristem size but not root patterning or root cell-type differentiation. In the gynoecium, *SPT* mediates the GCN5-CLV1 interaction, as *spt* mutants suppress multiple *clv1;gcn5* phenotypes, mostly by affecting auxin and cytokinin homeostasis and *WUS* expression [23]. However, no *SPT*-*GCN5* interaction has been reported in the root to date. To test whether *SPT* and *GCN5* interact genetically to control root meristem size and function, we created and analyzed *spt;gcn5* double mutants. Interestingly, in the double mutants, *spt* mutations partially suppressed *gcn5*-related defects in meristem size, meristem organization and root growth, suggesting that *SPT* interacts with *GCN5* in these aspects of root development.

## 2. Results

### 2.1. SPT Partially Rescues the gcn5 Root Growth Phenotype

**Figure 1:**
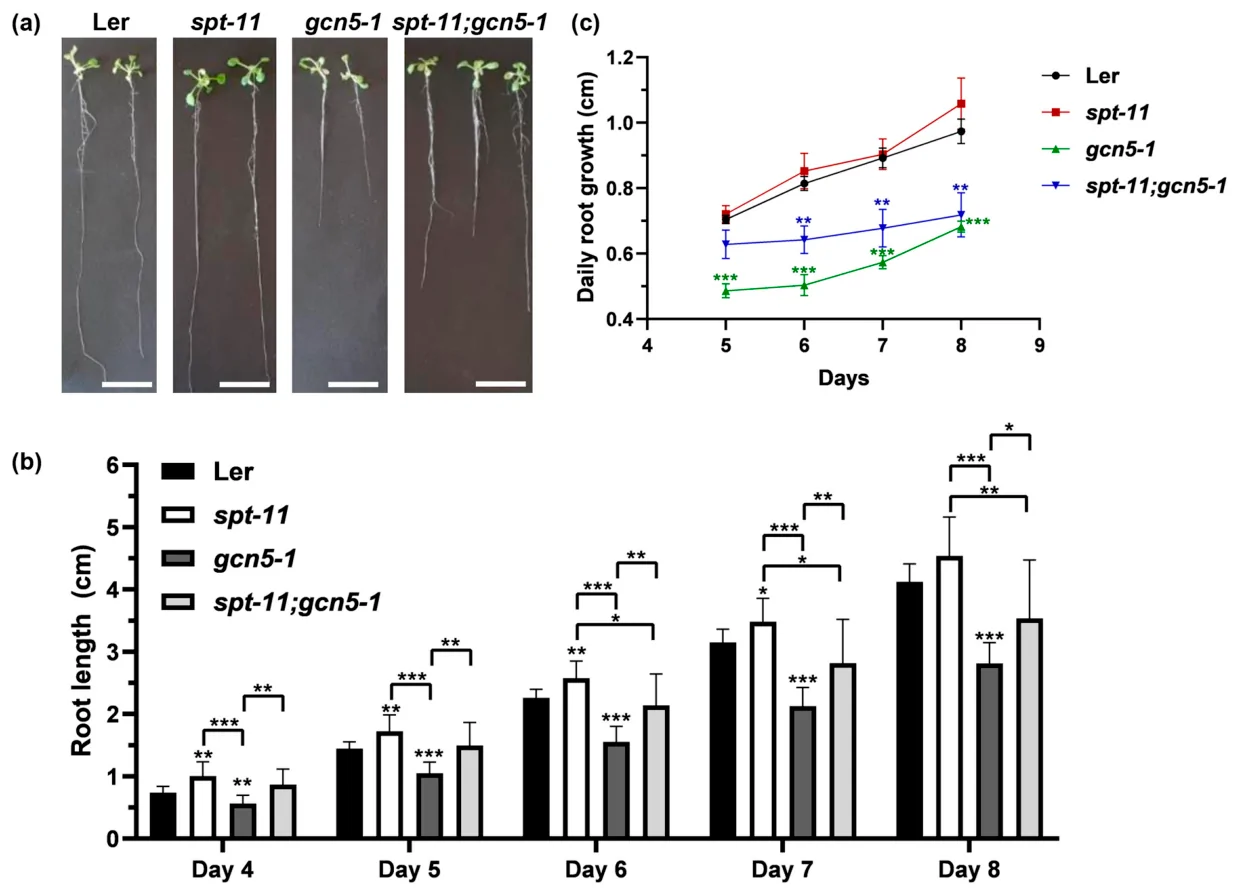
*GCN5* and *SPT* regulate root growth. (**a**) Phenotype of Landsberg erecta (Ler), *spt-11*, *gcn5-1* and *spt-11;gcn5-1* seedlings at 8 Days After Germination (DAG). Scale bar: 1 cm. (**b**) Bar plot of the primary root length in Ler, *spt-11*, *gcn5-1* and *spt-11;gcn5-1* seedlings between the 4th and 8th DAG. Error bars represent the standard deviation (SD) values. Statistical significance was calculated with two-way ANOVA with Geisser-Greenhouse correction and Tukey’s multiple comparisons test (* p < 0.05, ** p < 0.01 and *** p < 0.001). (**c**) Connected scatterplot of daily primary root growth in Ler, *spt-11*, *gcn5-1* and *spt-11;gcn5-1* seedlings between the 4th and 8th DAG. Error bars represent the standard error of the mean (SEM) values. Statistical significance was calculated with two-way ANOVA with Geisser-Greenhouse correction (** p < 0.01 and *** p < 0.001).

To analyze the possible genetic interaction of *GCN5* and *SPT* in the primary root, initially, root growth of *spt-11*, *gcn5-1* and *spt-11;gcn5-1* mutants were monitored daily for 8 days and were compared to that of wild-type roots. Root growth of *gcn5-1* was impaired compared to wild-type, whereas *spt-11* reached a longer root size at the end of the monitoring period (Figure 1a,b). The double mutant showed an intermediate daily root growth rate, resulting in a final root length between the single mutants and significantly longer than *gcn5-1* (Figure 1b,c).

### 2.2. GCN5 and SPT Regulate Meristem Size and QC Organization

**Figure 2:**
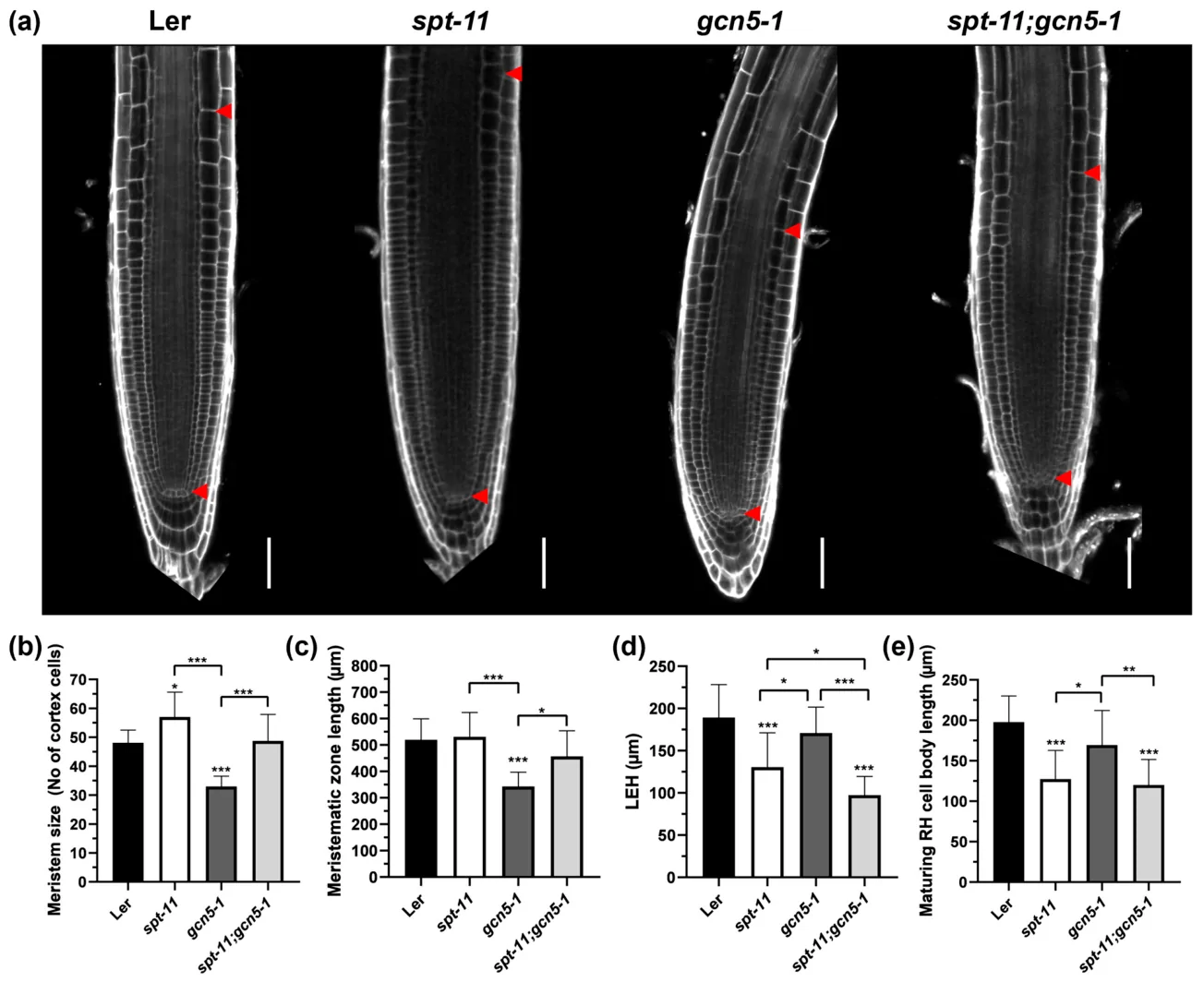
GCN5 and SPT regulate meristem size and cell elongation. (**a**) Confocal laser scanning microscope (CLSM) micrographs of the meristematic zone in Ler, *spt-11*, *gcn5-1* and *spt-11;gcn5-1* primary roots at 7 DAG. Red arrowheads indicate the QC and the first elongating cortex cell. Scale bar: 50 μm. (**b**,**c**) Bar plot of the root meristem size (**b**) and root meristem length (**c**) in Ler, *spt-11*, *gcn5-1* and *spt-11;gcn5-1* primary roots at 7 DAG. Root meristem size is expressed as the number of cortex cells in a file extending from the QC to the first elongated cortex cell [red arrowheads in (**a**)]. Error bars represent the standard deviation (SD) values. Statistical significance was calculated with a Brown-Forsythe and Welch ANOVA test with Games-Howell’s multiple comparisons test (* p < 0.05, and *** p < 0.001). (**d**,**e**) Bar plot of the cell body length of the 1st trichoblast (LEH) (**d**) and of a maturing root hair (RH) cell (**e**) in Ler, *spt-11*, *gcn5-1* and *spt-11;gcn5-1* primary roots at 7 DAG. Error bars represent the standard deviation (SD) values. Statistical significance was calculated with Brown-Forsythe and Welch ANOVA with Games-Howell’s multiple comparisons test (* p < 0.05, ** p < 0.01 and *** p < 0.001).

To test whether the root-length rescue resulted from a larger root apical meristem, propidium iodide (PI)-stained primary roots were observed under a confocal microscope, and meristem size (number of cortex cells) was measured. Consistent with the previously observed root lengths, *gcn5-1* had the smallest meristem size amongst the mutants, followed by *spt-11*;*gcn5-1* and, ultimately, by *spt-11* (Figure 2a,b). This was further supported by the total length of the meristematic zone (Figure 2c). Interestingly, *spt-11* and *spt-11*;*gcn5-1* cells elongated less than wild-type, while *gcn5-1* cell elongation remained unaltered (Figure 2d,e).

To determine whether the phenotypic effects of the *GCN5* and *SPT* genotypes are additive or interactive, we performed a two-way factorial ANOVA test incorporating their interaction term across all measured traits at 7 DAG. For primary root length and cell length (LEH), we found significant main effects of both genotypes (Table S1), but no significant interaction between the factors (*p* > 0.05). This might indicate that the genotypes act additively to regulate overall root length and cell elongation. In contrast, analysis of meristem size and meristem length revealed a statistically significant interaction between the two genotypes (*p*-values 0.0367 and 0.0251, respectively) (Table S1), further supporting a genetic interaction between *GCN5* and *SPT* at the root meristem level.

**Figure 3:**
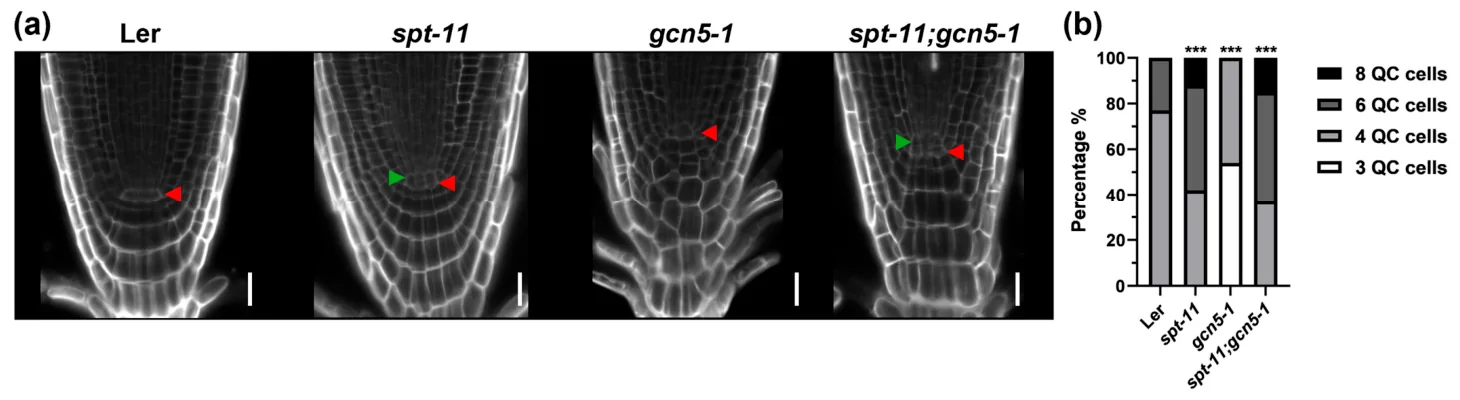
GCN5 and SPT regulate QC organization. (**a**) CLSM micrographs of the QC and root cap in Ler, *spt-11*, *gcn5-1* and *spt-11;gcn5-1* primary roots at 7 DAG. Red arrowheads indicate the QC, while green arrowheads indicate a second layer of QC cells. Scale bar: 20 μm. (**b**) Percentages of the measured QC cells in median longitudinal optical sections of CLSM micrographs in Ler, *spt-11*, *gcn5-1* and *spt-11;gcn5-1* primary roots at 7 DAG. Statistical significance from wild-type was calculated with a Chi-square test (*** p < 0.001). There is no statistical significance between *spt-11* and *spt-11;gcn5-1* (χ^2^: 1.447, df = 3, *p* > 0.05).

Additionally, the SCN organization was altered: *spt-11* had more than one layer of QC cells compared to wild-type (Figure 3). On the other hand, *gcn5-1* had aberrant QC and columella organization, whereas *spt-11;gcn5-1* strongly resembled the *spt-11* phenotype (Figure 3), suggesting that *SPT* is epistatic to *GCN5* in both QC size and organization.

### 2.3. GCN5 and SPT Modulate the Expression of Auxin- and Cytokinin-Related Genes

**Figure 4:**
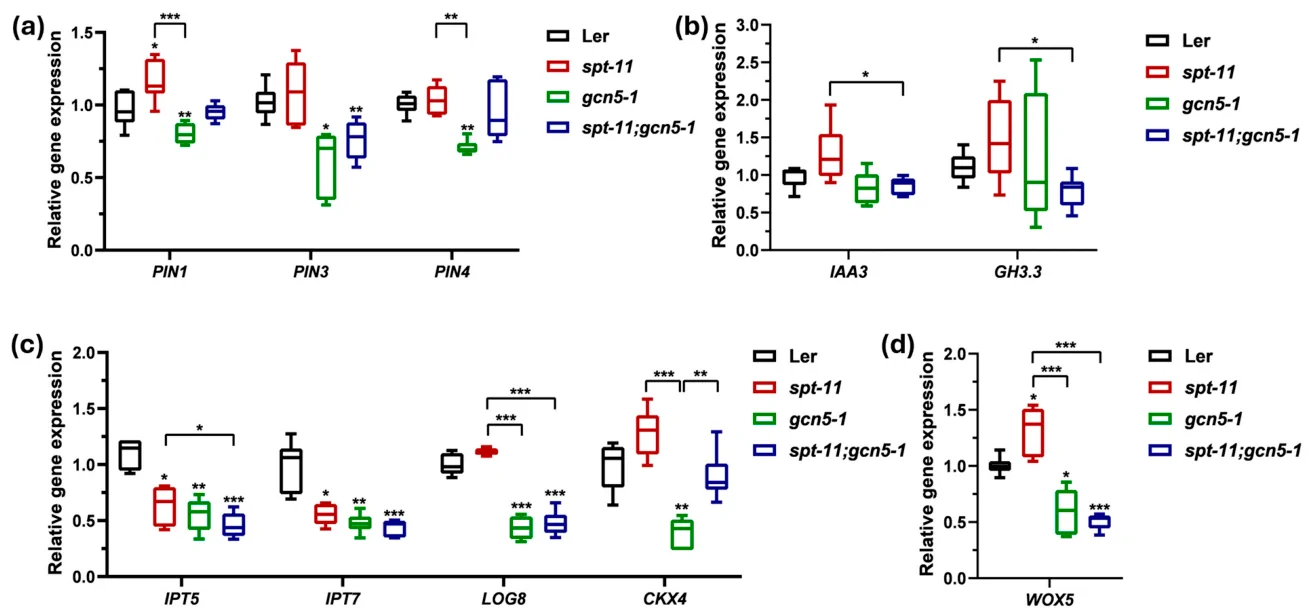
GCN5 and SPT regulate the expression of auxin- and cytokinin-related genes. (**a**–**d**) Boxplot of the relative expression of *PIN1*, *PIN3* and *PIN4* (**a**), *IAA3* and *GH3.3* (**b**), *IPT5*, *IPT7*, *LOG8* and *CKX4* (**c**) and *WOX5* (**d**) in Ler, *spt-11*, *gcn5-1* and *spt-11;gcn5-1* primary roots 7 DAG. The bars represent the min and max values, while the line represents the median. Statistical significance was calculated with Two-Way ANOVA with Geisser-Greenhouse correction and Tukey’s multiple comparisons test (**a**–**c**) and Brown-Forsythe and Welch ANOVA with Games-Howell’s multiple comparisons test (**d**) (* p < 0.05, ** p < 0.01 and *** p < 0.001).

Since GCN5 acts as a positive regulator of auxin distribution and cytokinin signaling in early root growth [18,19] and SPT regulates auxin accumulation/transport [22], we analyzed the expression of auxin- and cytokinin-related genes. We aimed to determine whether the observed morphological recovery in *spt-11;gcn5-1* was reflected at the gene expression level. In particular, we studied auxin transport and response alongside cytokinin biosynthesis and homeostasis. Only *PIN1* expression was increased in *spt-11*, whereas all *PIN-FORMED* (*PIN*) genes were downregulated in *gcn5-1*. The double *spt-11;gcn5-1* showed comparable *PIN1* and *PIN4* expression to wild-type levels (Figure 4a), but *PIN3* expression remained lower than wild-type, similar to *gcn5-1* (Figure 4a). In contrast, *INDOLE-3-ACETIC ACID INDUCIBLE 3* (*IAA3*) and *INDOLE-3-ACETIC ACID-AMIDO SYNTHETASE GH3.3* expression showed no major differences in mutants compared to wild-type (Figure 4b), whereas genes involved in cytokinin biosynthesis and homeostasis were clearly affected in the mutants. All three mutants showed reduced *ISOPENTENYLTRANSFERASE 5* (*IPT5*) and *ISOPENTENYLTRANSFERASE 7* (*IPT7*) expression compared to wild-type, while *LONELY GUY 8* (*LOG8*) down-regulation appeared to be *GCN5*-dependent (Figure 4c). Cytokinin homeostasis was also affected: *CYTOKININ OXIDASE 4* (*CKX4*) expression was restored to wild-type levels in *spt-11;gcn5-1*, compared with the reduced expression in *gcn5-1*. Finally, SPT and GCN5 functionally antagonize one another in the transcriptional regulation of *WOX5* expression, as *WOX5* was up-regulated and down-regulated in *spt-11* and *gcn5-1*, respectively. Although SPT and GCN5 act antagonistically to regulate *WOX5*, the double *spt-11;gcn5-1* mutant followed the transcriptional profile of *gcn5-1*, suggesting GCN5 has a more significant role in *WOX5* expression than SPT (Figure 4d).

## 3. Discussion

Both GCN5 and SPT regulate many facets of Arabidopsis development across several stages and tissues, including the root [17,21,22]. Their mutants exhibit pleiotropic phenotypes [14,15,20,22] with opposite effects in the root meristem, making them suitable for epistatic analysis. The *spt-11;gcn5-1* double mutants have longer primary roots with larger meristematic zones than *gcn5-1* single mutants, suggesting that *SPT* mutations partially suppress *gcn5* defects in root meristem development. In addition, *spt-11;gcn5-1* have larger QC than *gcn5*, with restored organization, suggesting that *SPT* is epistatic to *GCN5* for QC size and organization.

GCN5 and SPT have common target genes in the root, including *CYCB1;1*, which GCN5 promotes and SPT suppresses [15,22]. Restored expression levels of *CYCB1;1* or other cyclins in the double mutants could explain the restored cell proliferation in the root apical meristem. The homeobox root stem cell gene *WOX5* [24] is a potential GCN5 target for regulation through histone acetylation, at least in the context of callus formation [25]. It remains to be seen if GCN5 regulates *WOX5* through histone acetylation during normal root development. In our data, *WOX5* was significantly downregulated in *gcn5-1* mutants, consistent with the observed aberrant QC organization. In *spt-11* mutants, *WOX5* expression was upregulated, suggesting that SPT negatively regulates *WOX5* in the root. Interestingly, SPT has been shown to positively regulate *WUS* expression in the gynoecium style [23]. Since *WOX5* and *WUS* are closely related paralogous genes involved in maintaining stem cells in the root and shoot, respectively [24,26], it is not hard to envision SPT having a regulatory role in both developmental contexts. Whether *SPT* directly or indirectly affects *WOX5* is currently unknown. However, because *spt-11* mutants have more cells in the QC than WT roots, increased *WOX5* expression in *spt-11* roots could arise from the increased cell number. The *spt-11;gcn5-1* double mutant had downregulated *WOX5*, like *gcn5-1*, but more QC cells like *spt-11*. These data show that SPT and GCN5 both regulate *WOX5* expression, with GCN5 playing a more significant role than SPT. In addition, we suggest that *WOX5* transcript levels are not the primary reason for the differences in QC size and organization. If that were the case, the QC phenotype in double mutants with low *WOX5* expression should more closely resemble *gcn5-1*. SPT and GCN5 may regulate additional factors that affect QC.

Interestingly, SPT positively regulates cell elongation, while GCN5 does not seem to affect this process significantly. Since SPT affects both cell elongation and proliferation, its effect on total root growth becomes more prominent. SPT functions in the root meristem at late embryonic stages [22]. By contrast, GCN5 has been shown to function in the root meristem post-embryonically by regulating *PLT* gene expression [15]. *SPT*’s earlier function could explain its more significant role compared to *GCN5* in QC size and organization; in *spt;gcn5* mutants, the QC develops more cells even from the embryonic stage because of the loss of *SPT* function. Later, post-embryonically, when *GCN5* function is needed to promote QC size and proper organization, the QC already has more cells.

GCN5 and SPT could also affect the root meristem by modulating auxin and cytokinin homeostasis and responses. Both genes have been implicated in modulating auxin transport [18,22], affecting *PIN* expression and thus auxin output. *DR5* signal, suggesting active auxin signaling, has been shown to increase in *spt* mutants [22] and to be altered in *gcn5-1* mutants, with an increase in the QC and a reduction in columella cells [18]. Our results showed that *PIN* gene expression was reduced in *gcn5*, as expected [18], and restored in *spt;gcn5*; *PIN1* and *PIN4* expression was restored to wild-type levels, but *PIN3* remained downregulated. *SPT* has been known to regulate *PIN3* directly in flowers [27], but in the root, *PIN3* expression was unaltered in *spt* mutants, suggesting that a different regulatory mechanism may be in effect in different developmental contexts. *PIN1* expression was increased in *spt* mutants, suggesting that SPT has some regulatory role in auxin transport. SPT is an important positive regulator of cytokinin responses in the gynoecium [27], and GCN5 is known to affect them in the root [19]. Our expression analysis showed that *IPT5* and *IPT7*, two cytokinin biosynthesis genes, were downregulated in *spt* mutants and *CKX4*, a cytokinin catabolism gene, was somewhat upregulated, suggesting that cytokinin biosynthesis and cytokinin catabolism could be compromised. Interestingly, *gcn5* mutants also had downregulated *IPTs*, but they also had downregulated *CKX4*. They also exhibited significantly downregulated *LOG8* expression, suggesting that GCN5 promotes cytokinin biosynthesis in the root at different biosynthesis steps. It could be hypothesized that the reduced cytokinin biosynthesis in *gcn5* could be counterbalanced by the reduced *CKX4*-dependent catabolism, but in *spt* mutants, the increased catabolism could further deplete cytokinin levels.

A genetic framework has been proposed for the hormonal regulation of root development [28,29]. Briefly, in the transition zone, cytokinin acts to antagonize auxin responses. Through AHK3-ARR1-dependent *SHY2/IAA3* expression, cytokinin negatively regulates *PIN* expression, thereby altering auxin distribution. In addition, *SHY2*/*IAA3* suppresses auxin-induced *IPT5* expression, negatively regulating auxin-induced cytokinin response. SHY2/IAA3 protein is negatively regulated by auxin [28,30].

In this framework, GCN5 affects both auxin and cytokinin responses. *GCN5* positively regulates *PIN*s, enhancing the proper spatio-temporal distribution of auxin. *GCN5* also positively regulates *IPT5*, suggesting it promotes cytokinin biosynthesis. SPT also contributes to the regulation of auxin and cytokinin responses. It negatively affects *PIN1* expression and has been known to negatively affect PIN4 protein expression and localization [22]. Although *SHY2*/*IAA3* transcript levels were not altered in the mutants compared to wild-type, it remains unknown whether SPT or GCN5 affects SHY2/IAA3 protein abundance or activity. SPT also positively regulates the expression of the cytokinin biosynthesis gene *IPT5*. Taken together, these findings indicate that GCN5 and SPT may modulate root development by coordinately regulating auxin transport and cytokinin biosynthesis, thereby influencing the hormonal balance that governs the transition between distinct developmental zones of the root. A detailed analysis of auxin and cytokinin distribution in *spt*, *gcn5*, and *spt;gcn5* double mutants with reporter genes, complemented with direct measurements of hormone levels, could reveal the synergy between GCN5 and SPT in auxin and cytokinin responses in the root.

One limitation of this study is that gene expression analyses were performed on whole-root samples, even though many of the genes examined are expressed in specific subsets of root cells or tissues. Because the mutant lines exhibit alterations in root architecture, some observed transcriptional differences may reflect changes in the relative abundance or spatial distribution of specific cell types rather than cell-autonomous changes in gene expression.

## 4. Materials and Methods

### 4.1. Plant Material and Growth Conditions

*Arabidopsis thaliana* (L.) Heynh. *spt-11*, *gcn5-1* and *spt-11*;*gcn5-1* were previously described [23] and were introgressed into the Ler (Landsberg erecta) genetic background. Seeds were surface sterilized in 30% *w*/*v* commercially available bleach solution for 10 min, stratified for 2 days at 4 °C and sown on plates with Gamborg B5 medium (Duchefa, Amsterdam, Netherlands), supplemented with 1% *w*/*v* sucrose (Duchefa, Amsterdam, Netherlands) and 0.8% *w*/*v* Phytoagar (Duchefa, Amsterdam, Netherlands), with pH adjusted to 5.7. Plants were grown at 20 ± 2 °C, under 80 μmol m^−2^ s^−1^ cool-white and fluora fluorescent lighting (Osram, Premstaetten/Graz, Austria) and long-day conditions (16 h light, 8 h darkness).

### 4.2. Genetic Analysis

Because *gcn5-1* and *spt-11*;*gcn5-1* mutant plants could not produce seeds in the Ler genetic background, they were maintained as a heterozygous population (*gcn5-1/+* and *spt-11;gcn5-1/+*). At the end of each experiment, we extracted DNA from whole seedlings as described in [31] and genotyped them for the GCN5 and SPT genes and the *gcn5-1* and *spt-11* T-DNAs using the primer pairs in Table S2. Only measurements from homozygous mutants were kept for visualization and statistical analysis.

### 4.3. Root Growth Assay

Primary root length in vertically grown seedlings was monitored daily from the 4th day after germination (DAG) up until the 8th day. Seedlings (*n* = 15) were observed with a Zeiss SteREO Discovery.V8 stereomicroscope equipped with a Zeiss AxioCam ERc 5s (Carl Zeiss AG, Munich, Germany). Fiji V1.54 was used to measure primary root length. The daily root growth rate was calculated as the difference in length between consecutive days.

### 4.4. Root Meristem Size Analysis

The root tips of seedlings (7 DAG) were counterstained with propidium iodide (PI; 20 μg/ml in phosphate-buffered saline (PBS)) and examined with a confocal laser scanning microscope (CLSM) using a Zeiss Observer.Z1 inverted microscope equipped with an LSM780 module (Carl Zeiss AG, Munich, Germany). PI was excited at 543 nm and detected at 580–650 nm. Specimens (*n* = 13) were observed with either a Plan-Apochromat 25x/0.8 NA objective or a Plan-Apochromat 40x/1.3 NA oil-immersion objective, and analysis was performed with Zeiss Zen 3.13 (Zen lite, Blue version). For meristem size analysis, the length of the meristematic zone in Fiji V1.54, the number of cortex cells between the QC and the first elongating cortex cell were calculated [32], and the number of QC cells.

### 4.5. Cell Size Analysis

The root tips of seedlings (7 DAG) were observed with a Zeiss AxioImager.Z2 microscope equipped with a Zeiss AxioCam MRc 5 (Carl Zeiss AG, Munich, Germany) and the maturation zone was photographed. The cell body length of the first epidermal cell with a visible root hair bulge (LEH) was measured (*n* = 20) in Fiji V1.54, along with the cell body length of more mature root hair cells (*n* = 30) [33].

### 4.6. Gene Expression Analysis (RT-qPCR)

For the RT-qPCR expression analysis, roots from 7 d-old seedlings were excised from the above-ground tissue. Each root and above-ground part was collected separately into 1.5 mL tubes and flash-frozen in liquid nitrogen. The above-ground part was used for genotyping as described previously, and 15 roots of Ler, *spt-11*, *gcn5-1*, and *spt-11*;*gcn5-1* were pooled into one tube. Three biological replicates were performed, and total RNA was extracted using the GF-1 Total RNA Extraction Kit (Vivantis Technologies, Subang, Malaysia). RNA quality and quantity were assessed by 1% agarose gel electrophoresis and spectrophotometrically, using the NanoDrop 2000 (Thermo Fisher Scientific, Waltham, MA, USA). PrimeScriptTM 1st strand cDNA synthesis kit (Takara, Shiga, Japan) was used for reverse transcription. Reverse transcription was performed using 100 ng of total RNA. Quantitative reverse-transcription polymerase chain reactions (RT-qPCRs) were prepared with the Xpert Fast SYBR (GRISP Research Solutions, Porto, Portugal) kit using the ABI StepOnePlus™ system (Applied Biosystems, Foster City, CA, USA). Three technical replicates for each sample were performed, and the *RHIP1* (AT4G26410) gene was used as an endogenous control (Table S2). Data were analyzed with the ΔΔCt method using StepOne Software 2.1 (Applied Biosystems, Foster City, CA, USA).

### 4.7. Data Visualization and Statistical Analysis

All data visualization and statistical analyses were performed using GraphPad Prism 8.0.1. For root length, statistical significance was calculated with two-way ANOVA with Geisser-Greenhouse correction and Tukey’s multiple comparisons test. For daily root growth, we used two-way ANOVA with Geisser-Greenhouse correction. For meristem size and meristematic zone length, statistical significance was calculated with Brown-Forsythe and Welch ANOVA with Games-Howell’s multiple comparisons test. For cell length analysis, we used Brown-Forsythe and Welch ANOVA with Games-Howell’s multiple comparisons test. For QC size analysis, statistical significance was calculated with a Chi-square test. For gene expression analysis, statistical significance was calculated with Two-Way ANOVA with Geisser-Greenhouse correction and Tukey’s multiple comparisons test and Brown-Forsythe and Welch ANOVA with Games-Howell’s multiple comparisons test. To determine whether the *GCN5* and *SPT* genotypes act independently or interactively, data for primary root length, cell length (LEH), meristem size and meristem length were analyzed using a two-way factorial ANOVA. The main effects of genotype *GCN5*, genotype *SPT* and their interaction term were included.

## 5. Conclusions

**Figure 5:**
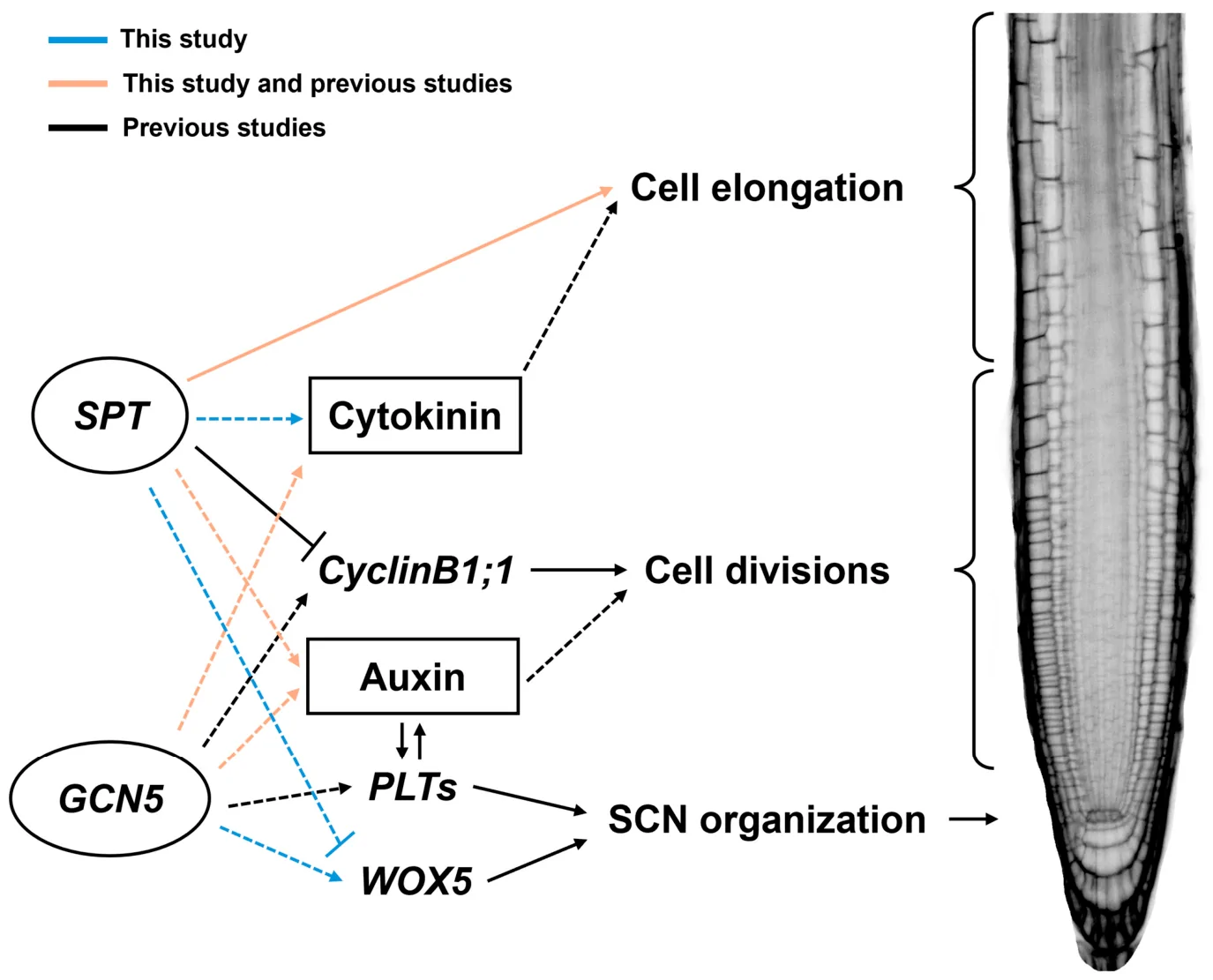
Proposed model for the regulation of SCN maintenance, cell divisions and cell elongation by GCN5 and SPT. Line color indicates which data are derived from this study (blue lines) or from previously published studies (orange and black lines). Dashed lines depict either direct or indirect regulation.

Based on our results and published data, we propose the following model (Figure 5). Both SPT and GCN5 affect cell proliferation and SCN organization in the root of *Arabidopsis thaliana.* SPT is also a positive regulator of cell elongation and could influence this process by regulating cytokinin biosynthesis. Its negative regulation of *CYCB1;1* expression shows how SPT could control cell division in the meristematic zone. SPT could also affect cell division through its regulatory role on auxin homeostasis, especially through the expression of auxin transport genes. SPT influences SCN organization, at least in part, by its restrictive role on *WOX5* expression. GCN5 also positively regulates cytokinin biosynthesis genes, though its effect on cell elongation is less obvious. GCN5 positively regulates *CYCB1;1* expression and could, through this, control cell division, alongside its significant role in the expression of auxin homeostasis genes. For SCN organization, GCN5 positively regulates the PLT pathway and promotes *WOX5* expression in the root.

It is unknown whether SPT and GCN5 interact physically or act together at the same gene loci to control transcription. This model suggests that SPT and GCN5 either directly or indirectly regulate key factors involved in specific root functions, like *WOX5* in SCN organization, *CYCB1;1* and other cyclins in cell proliferation and hormone biosynthesis, transport or catabolism genes, creating a complex genetic interaction that governs root meristem size and organization and, ultimately, root growth.

## Data Availability

All data are available upon reasonable request.

## Supplementary Materials

The following supporting information can be downloaded at: https://www.mdpi.com/article/10.3390/plants15203181/s1. Table S1. Two-way ANOVA statistical summary of the main and interactive effects of genotype *GCN5* and genotype *SPT* on the measured root traits. Table S2: List of primers used in this study.

## Abbreviations

The following abbreviations are used in this manuscript:

bHLH	basic Helix-Loop-Helix
CKX4	CYTOKININ OXIDASE 4
CLSM	Confocal Laser Scanning Microscope
CYCB1;1	CYCLINB1;1
CYCB1;3	CYCLINB1;3
DAG	Days After Germination
GCN5	GENERAL CONTROL NON-DEREPRESSIBLE 5
GH3.3	INDOLE-3-ACETIC ACID-AMIDO SYNTHETASE GH3.3
IAA3	INDOLE-3-ACETIC ACID INDUCIBLE 3
IPT5	ISOPENTENYLTRANSFERASE 5
IPT7	ISOPENTENYLTRANSFERASE 7
LEH	Cell body length of the first epidermal cell with a visible root hair bulge
Ler	Landsberg erecta
LOG8	LONELY GUY 8
PBS	Phosphate-Buffered Saline
PI	Propidium Iodide
PIN	PIN-FORMED
PLTs	PLETHORAs
QC	Quiescent Center
RAM	Root Apical Meristem
RH	Root Hair
SCN	Stem Cell Niche
SCR	SCARECROW
SHR	SHORT-ROOT
SPT	SPATULA
WOX5	WUSCHEL-RELATED HOMEOBOX 5
