# Supplementary material for: Loss of SPATULA Function Partially Suppresses Root Growth and Meristem Organization Defects in GCN5 Mutants in Arabidopsis thaliana
Source: Plants (Basel). 2026 Oct 16;15(20):3181. doi: 10.3390/plants15203181 (PMC13709297; doi:10.3390/plants15203181)
Supplement: Supplementary file 1 [file plants-15-03181-s001.zip › plants-4599918-supplementary.pdf]

Supplementary materials

Table S1. Two-way ANOVA statistical summary of main and interactive effects of genotype *GCN5* and genotype *SPT* on the measured root traits. Values show F-statistics (degrees of freedom) and associated P-values for the main effects of each genotype and their interaction term.

**Trait**	**Genotype GCN5**<br>F (DFn, DFd)<br>P-value	**Genotype SPT**<br>F (DFn, DFd)<br>P-value	**Interaction**<br>F (DFn, DFd)<br>P-value	**Interpretation**
**Primary root length**	F (1,56) = 59.63<br>P < 0.0001	F (1,56) = 16.92<br>P = 0.0001	F (1,56) = 1.870<br>P = 0.176	Additive effect
**LEH**	F (1,69) = 9.530<br>P = 0.0029	F (1,69) = 68.32<br>P < 0.0001	F (1,69) = 1.462<br>P = 0.2307	Additive effect
**Meristem size**	F (1,48) = 39.39<br>P < 0.0001	F (1,48) = 50.92<br>P < 0.0001	F (1,48) = 4.620<br>P = 0.0367	Significant interaction
**Meristem length**	F (1,48) = 32.13<br>P < 0.0001	F (1,48) = 7.578<br>P = 0.0083	F (1,48) = 5.347<br>P = 0.0251	Significant interaction

Table S2. List of primers used in this study.

Accession Number	Gene Name		Primer sequence (5'→ 3')	Purpose
AT3G54610	*GCN5*	F	AGTGGGGGCACACTCGTTTCAAATTATTC	Genotyping
		R	TTGAGATTTAGCACCAGATTGGAGACCTG	
	gcn5-1 T-DNA	F	CATTTTATAATAACGCTGCGGACATCTAC	
		R	TTGAGATTTAGCACCAGATTGGAGACCTG	
AT4G36930	*SPT*	F	CTGACACAAGCTCATTGCTA	
		R	TTCACTCAAAATCACAACCA	
	spt-11 WiscDs	F	CTGACACAAGCTCATTGCTA	
		R	AACGTCCGCAATGTGTTATTAAGTTGTC	
AT4G26410	*RHIP1*	F	GAGCTGAAGTGGCTTCCATGA	RT-qPCR
		R	GGTCCGACATACCCATGATCC	
AT1G73590	*PIN1*	F	CGTTTGTGTTTGCCAAACAGT	
		R	AACAAAGTCCCTGTGTTTTGGT	
AT1G70940	*PIN3*	F	GAGCACCTGACAACGATCAAG	
		R	TCCACTTGCTGGATGAGCTAC	
AT2G01420	*PIN4*	F	TGCTAAGGAGATTCGGATGG	
		R	AAGACCGCCGATATCATCAC	
AT1G04240	*IAA3*	F	AAAGGCTCAGATTGTTGGATGGC	
		R	TGACCCTCATGCTCAGATTCATTC	
AT2G23170	*GH3.3*	F	CATCACAGAGTTCCTCACAAGC	
		R	GTCGGTCCATGTCTTCATCA	
AT5G19040	*IPT5*	F	GATGACCACCATTTGGAAGG	
		R	TGTTTTACCTCACCGGGAAA	
AT3G23630	*IPT7*	F	GACCAAGCGAGAGAATCGTC	
		R	GTAAGATGCCGCCAAACAGT	
AT5G11950	*LOG8*	F	TTGTGGTTTCTGCTCCAACA	
		R	CCGGGTAATCTCCGAGTTCT	
AT4G29740	*CKX4*	F	ATCCCACAAACCGAAACAAA	
		R	TCCACCAGCTGATTGCAGTA	
AT3G11260	*WOX5*	F	CGTGGCAACAATAACGGAGG	
		R	CTAAGACCGGCTCGAAACAGA	
